# Chemical, Functional, and Technological Features of Grains, Brans, and Semolina from Purple and Red Durum Wheat Landraces

**DOI:** 10.3390/foods11111545

**Published:** 2022-05-25

**Authors:** Afef Ladhari, Giandomenico Corrado, Youssef Rouphael, Francesca Carella, Giuseppina Rita Nappo, Cinzia Di Marino, Anna De Marco, Domenico Palatucci

**Affiliations:** 1 Laboratoire GREEN TEAM (LR17AGR01), Institut National Agronomique de Tunisie (INAT), Université de Carthage, 43 Avenue Charles Nicolle, Tunis 1082, Tunisia; afef.ladh@yahoo.fr; 2 Department of Agricultural Sciences, University of Naples Federico II, Via Università 100, 80055 Portici, Italy; giandomenico.corrado@unina.it (G.C.); youssef.rouphael@unina.it (Y.R.); 3 Department of Biology, University of Naples Federico II, Via Cinthia 21, 80126 Naples, Italy; francesca.carella@unina.it; 4 Sannio Tech Consortium, Piazza San G. Moscati 5, 82030 Apollosa, Italy; pinanappo@gmail.com; 5 Department of Chemical Sciences, University of Naples Federico II, Via Cinthia 26, 80126 Naples, Italy; cdimarin@unina.it; 6 Department of Pharmacy, University of Naples Federico II, Via Montesano 49, 80131 Naples, Italy; anna.demarco@unina.it

**Keywords:** durum wheat, diversity, pigmented cereals, phytochemicals, anthocyanins, antioxidant activity, protein, gluten

## Abstract

A main reason of the increasing interest in cereal landraces is their potential to offer more diversified and functional staple food. For instance, landraces are an underexploited resource of pigmented varieties, appreciated for the high accumulation of phytochemicals with known health benefits. This study characterized the chemical, functional, and technological features of the bran, semolina, and grains of two durum wheat (*Triticum turgidum* L. subsp. *durum*, Desf.) landraces, named ‘Purple’ and ‘Red’ for their grain color, collected in Ethiopia and grown and sold in southern Italy as a niche product. Specifically, we analyzed the protein content, dry gluten, ash, total polyphenols, anthocyanins, proanthocyanidins, and specific phenolic acids. We also evaluated the antioxidant activity using DPPH- and ABTS-based methods. The two landraces had positive nutritional features, such as a high protein content, a rich and composite range of secondary metabolites (which include specific phenolic acids and anthocyanins), and antioxidant activities in all the fractions analyzed. The germplasm under investigation therefore has a well-justified potential to yield functional products and to diversify durum wheat-based foods.

## 1. Introduction

Wheat is one of the first domesticated cereal plants and it has been globally cultivated for its grains since the dawn of civilization. In the last decade, its world production has increased, currently reaching 750 million tons [1], while the sowing area has fluctuated around 220 million hectares. China (17%) and India (12%) are the top producers and collectively the European Union produces around 15% of the world’s total. In Italy, about 2 million hectares are cultivated, prevalently with durum wheat, for a production of 8 million tons [2]. The yield increase in the last century is the joint result of different factors, with plant breeding having a significant role in shaping the morphological and technological features of contemporary varieties [3]. These are characterized by a reduced height, a more efficient assimilate partitioning, diminished sensitivity to photoperiod, adaptability to certain agronomic conditions, and resistance to specific races of fungal pathogens [3], traits that are expected to be absent in the old varieties of wheats [4].

Durum wheat (*Triticum turgidum* L. subsp. *durum*, Desf.) is a tetraploid species (AABB; 2n = 4x = 28) with better tolerance to drought and heat than the hexaploid common wheat (*Triticum aestivum* L; AABBDD; 2n = 6x = 42). Durum wheat, also known as hard, pasta, or macaroni wheat, is mainly cultivated in Mediterranean countries, North America, Argentina, and eastern Europe. In Italy, the production is principally located in southern regions such as Apulia and Sicily. Durum wheat is central to the gastronomy of Mediterranean countries because it is employed to produce pasta and couscous, as well as bulgur, puddings, pastries, freekeh, kishk, and other traditional dishes. An important aesthetic and commercial feature of durum wheat semolina is the color. The typical yellow-amber pigmentation is predominantly due to lipophilic carotenoids within the kernel, mainly lutein [5]. Nonetheless, anthocyanins are also another class of pigmented phytochemicals that can be present in high amount in the grains of some wheat varieties. According to the quantity and type, this class of water-soluble pigments can give rise to wheat grains with colors ranging from red to purple [5].

In recent years, the scientific interest and appreciation of the quality of traditional wheat varieties has increased for a more sustainable low-input production of grain, as germplasm with an enhanced phytochemical profile, and as a source of adaptive traits in the face of climate change [6,7]. For instance, considering that wheat is a staple food in several countries, anthocyanin-rich grains can be used to produce a wide range of foods with enhanced nutraceutical and pharmaceutical properties. Old varieties are also gaining popularity to satisfy consumer demand for regional crop production and food manufacturing, to diversify the dietary basket, and to provide commercially novel products richer in health-promoting ingredients [7,8]. Regrettably, the compositional properties of anthocyanin-rich grains of landraces, as well as old durum wheat varieties, have not yet been fully acknowledged, not only if compared with soft wheat varieties, but also with old species such as einkorn, emmer, and spelt [5,9,10,11,12].

The aim of this research was to explore the chemical, functional, and technological features (such as the contents of proteins; dry gluten; gluten index; ash; total polyphenols; antioxidant activity) of two differently pigmented *Triticum turgidum* landraces. Moreover, we quantified major anthocyanins (Delphinidin 3-glucoside, Delphinidin 3-rutinoside, Cyanidin 3-glucoside, Petunidin 3-glucoside, Peonidin 3-glucoside, and Malvidin 3-glucoside), anthocyanidins (i.e., cyanidin, delphinidin, malvidin, peonidin, and petunidin), and specific phenolic acids (ferulic acid, *p*-hydroxybenzoic acid, vanillic acid, and *p*-coumaric acid). These two landraces were named ‘Purple’ and ‘Red’ according to the color of the grain and were originally collected from the Oromya region, one of regional states of Ethiopia. Cereal landraces are mainly evaluated as a source of inheritable traits that may favor local adaptation and productivity in sustainable agriculture [13], but they also have desirable characteristics related to food quality and nutritional benefits. Therefore, our detailed characterization contributes to demonstrate the value of durum wheat landraces as a rich source of primary and secondary metabolites of interest for human health and nutrition.

## 2. Materials and Methods

### 2.1. Materials

We analyzed two durum wheat (*Triticum turgidum* L. subsp. *durum*, Desf.) landraces named ‘Purple’ and ‘Red’. They are maintained and multiplied by Agrismarter (Foggia, Italy), marketed by the company Granomischio (Foggia, Italy), and cultivated in areas near the Daunian Mountains (Apulia region, Italy). Grains (i.e., intact caryopses), bran (i.e., kernel components except the flour fraction at the given extraction rate, ~65–75%), and semolina were provided also by the Granomischio company in September 2021. For their analysis, grains were ground to a fine powder using a blender stored at −20 °C. Semolina and bran were processed by a professional milling company with a multi-pass roller system. Briefly, grains were purified (mainly to remove low-density particles), surface cleaned (to remove impurities and possible abiotic and biotic contamination), pearled/scoured, milled, and purified/sieved to semolina.

Chemicals, analytical grade reagents, and standards of phenolic compounds were obtained from Sigma-Aldrich (Milan, Italy).

### 2.2. Determination of Dry Gluten

Gluten was extracted from semolina (*n* = 5) according to previously described procedure [14]. Twenty grams of semolina were suspended in 12.5 mL of a 4% monosodium/disodium phosphate buffer at pH of 6.8, diluted to a ratio of 1:40 with a 2% NaCl solution. After 30 min, the dough was washed using a Glutomatic System 2200 (Perkin Elmer, Turin, Italy) with the NaCl solution to remove soluble proteins and starch. The pure gluten obtained was dried in an oven (Heraeus T6200, Progitec, Sabaudia, LT, Italy). The gluten content was expressed as dry gluten per 100 g of material.

### 2.3. Determination of Gluten Index

The determination of the gluten index comprised three steps: (i) the gluten extraction and quantification of the wet gluten; (ii) the centrifugation of the wet gluten; and (iii) the calculation of the gluten index, according to standard procedures [14] using a Gluten Index 2100 centrifuge (Bastak, Ankara, Turkey). The gluten index is the percentage ratio of the wet gluten remaining on the sieve (after centrifugation) to the total wet gluten.

### 2.4. Determination of Total Protein Content

Total proteins were quantified using the Kjeldahl method. Briefly, samples (1 g) were digested in 15 mL of 98% H_2_SO_4_ in the presence of a catalyst (K_2_SO_4_:CUSO_4_, 9:1 *w/w*). Then, 50 mL of 40% NaOH (*w*/*v*) was added to covert the released ammonium into ammonia, which was distilled and collected in a flask containing a known amount of excess acid (0.1 M HCl). The excess acid was back-titrated with 0.1 M NaOH. The protein content refers to 100 g of substance and is calculated using the following formula:

Proteins (%) = [100 × (V_NaOH_ × C_NaOH_ − V_HCl_ × C_HCl_) × 14.0067 × 5.70]/g

where V_NaOH_ are the liters of NaOH; C_NaOH_ is the molar concentration of NaOH; V_HCl_ are the liters of HCl; C_HCl_ is the molar concentration of HCl; 14.0067 is the atomic weight of nitrogen; 5.70 is the conversion factor for proteins; and g is the weight of the sample in grams [15].

### 2.5. Determination of Ash

The quantification of ash was performed essentially as described [16]. Briefly, a weighed sample of semolina (5–10 g) was placed in a platinum capsule and heated at 550–590 °C in the muffle furnace Srefo R-1905 (Zhuhai Refine Zhizao, Guangdong, China) until light gray ash was obtained (5 h). The weight of the ash refers to 100 g of dry matter. The measurement was carried out on five replicates.

### 2.6. Phenolic Extraction

**Figure 1:**
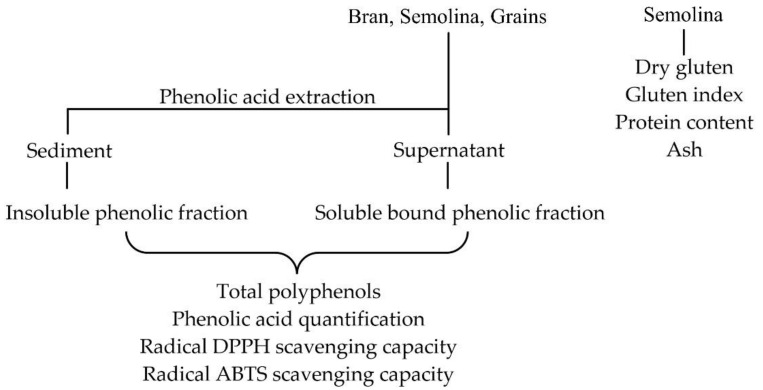
Flowchart of the process used for the extraction and analysis of the wheat material.

The phenolic extracts were prepared as reported [17]. The steps of the approach are summarized in Figure 1. About 1.5 g of wheat material was pulverized and suspended in 30 mL of a methanol/hydrochloric acid solution (99:1, *v*/*v*). The mixture was stirred for 30 min at room temperature. The suspension was then centrifuged (PK121R Multispeed, ALC International, Milan, Italy) at 10,000 rpm for 5 min at room temperature. The sediment was extracted five times.

### 2.7. Soluble Phenolic Fraction

Two milliliters of the acidified methanolic extract were dried using a rotavapor (Rotavapor RE 111, Buchi, Switzerland) at 30 °C. The dried material was then suspended in 20 mL of 3M KOH and stirred for about three hours at room temperature. The solution was acidified with 1M HCl to pH 3 and extracted three times with a 1:1 (*v*/*v*) mixture of petroleum ether/ethyl acetate. Extracts were treated with anhydrous sodium sulfate, filtered on a Whatman paper Grade 1, dried under a light stream of nitrogen, and resuspended in methanol.

### 2.8. Insoluble Bound Phenolic Fraction

The sediment (Figure 1) was suspended in 20 mL of 3M KOH at room temperature for about three hours, under continuous stirring, acidified with 1M HCl to pH 3, and extracted three times with a 1:1 (*v*/*v*) petroleum ether/methylene chloride solution. Extracts were treated as in paragraph 2.2 and resuspended in methanol. All extractions were performed avoiding direct light to minimize photo-oxidation.

### 2.9. Total Polyphenols

The total phenol content in the soluble and insoluble bound extracts and in the different organic extracts (Figure 1) was quantified with the Folin–Ciocalteu method using gallic acid as a standard as previously reported [18]. The extracts were solubilized in 5 mL of methanol, with the aid of a sonicator. One milliliter was taken and filtered on a Phenex filter (0.45 µm) and diluted to a 10 mL final volume. Samples (*n* = 5) and solutions of the standard were tested with the colorimetric method and absorbance was read at 765 nm [19]. Specifically, 0.2 mL of the solution of the sample to be analyzed (or of the standard, or Milli Q water, in the case of the blank) was added with 0.8 mL of Milli Q water and 0.2 mL of the Folin–Ciocalteu reagent. The solution was incubated for 5 min and then another 0.8 mL of Milli Q water and 2 mL of an aqueous 8% Na_2_CO_3_ solution were added. A standard curve was built with gallic acid at the 4.8, 9.6, 48, 96, 240, and 480 µg/mL concentrations. The minimum threshold for accepting the calibration curve was r = 0.97. Results were expressed as milligrams of gallic acid equivalents.

### 2.10. Phenolic Acid Quantification by HPLC

Both phenolic extracts (Figure 1) were analyzed by HPLC in accordance with the already published protocols [20,21]. A Shimadzu LC-8A HPLC instrument (Shimadzu, Milan, Italy) was used with a 2.6 mm 100 Å (100 × 4.6 mm) Kinetex reverse phase column. The eluent phase consisted of a mixture of A (2% AcOH in water, *v*/*v*) and B (methanol), with a constant flow of 1.2 mL/min and a wavelength of the UV detector set at 280 nm. The gradient, in terms of eluent B, was 10% at time 0, 20% at 10 min, 25% at 15 min, and 30% at 30 min. The presence of ferulic acids, *p*-hydroxybenzoic acid, and vanillic and coumaric acids was measured through the corresponding calibration lines obtained from the corresponding standard samples commercially available from Sigma-Aldrich. The calibration curves were linear in the concentration intervals considered. In particular, the detection limits were equal to 12, 0.08, 0.1, and 1.11 µg/mL, respectively, for the four aforementioned phenolic acids. The analyses were performed five times; the results were expressed as micrograms per kilogram of wheat on a dry matter (DM) basis.

### 2.11. Determination of Anthocyanins Content

Total anthocyanin extract was prepared essentially as reported [22] and quantification was carried out with a spectrophotometric method [23], using catechin as standard with concentration ranging from 2 to 200 µg/mL. Results were expressed as µg catechin equivalents/g dry weight material. The equation obtained from the standard curve is y = 0.0018x + 0.0146, where y is absorbance at 535 nm and x is concentration of catechin standard.

### 2.12. Determination of Proanthocyanidin Content

Proanthocyanidin extract was prepared using previously published procedures [23] and the proanthocyanidin content was determined also as already described [24], using catechin as standard with concentrations ranging from 100 to 1000 µg/mL. Results were expressed as µg catechin equivalents/g dry weight material. The equation obtained from the standard curve is y = 0.0023x + 0.0187, where y is absorbance at 510 nm and x is concentration of catechin standard.

### 2.13. Radical DPPH Scavenging Capacity

The DPPH^.^ antioxidant activity of the material under investigation (Figure 1) was evaluated using already published procedures [25] with minor modifications. One milliliter of extraction solvent with different extract dilutions was added to two mL of DPPH in methanol (5 × 10^−5^ M). The reaction was carried out at 25 °C for 30 min. After half an hour, the absorbance value reached a constant value, which was used to calculate the percentage of residual DPPH. Radical reduction by antioxidants was monitored by measuring the absorbance at 517 nm using a Perkin Elmer Lambda 7 spectrophotometer (Beckman, Brea, CA, USA). Five extracts were analyzed for each sample, each with four different dilutions. A regression line was also calculated for the reference antioxidant Trolox, with concentrations ranging from 3 to 50 μM. The antioxidant activity was expressed as the ratio between the I_50_ of Trolox and the I_50_ of the sample, that is, micromoles of Trolox equivalent (TE) per gram of DM.

### 2.14. Radical ABTS Scavenging Capacity

The ABTS antioxidant activity (Figure 1) was evaluated as already published [26], quantifying the ability of natural extracts to convert the radical cation ABTS^+^, generated from the corresponding acid using as oxidizing agent sodium or potassium persulfate (K_2_S_2_O_8_ or Na_2_S_2_O_8_) in its neutral form. For the ABTS assay, the antioxidant capacity was also expressed as the Trolox equivalent antioxidant capacity (TEAC), a unit of measurement defined as the quantity of Trolox needed to obtain the same antioxidant activity as the sample (micromoles of TE per g of sample).

### 2.15. Statistical Analysis

Data are reported as mean value ± standard deviation (SD). The normality of the data distribution was assessed by the Shapiro-Wilk test. The independent Student’s *t*-test was employed for mean separation considering as the threshold of statistical significance a *p*-value lower than 0.05. Calculations were performed with the SigmaPlot 12.2 software (Systat Software, San Jose, CA, USA).

## 3. Results and Discussion

### 3.1. Analysis of Dry Gluten, Gluten Index, Protein Content, and Ash

The two wheat varieties analyzed had a similar percentage of dry gluten, around 12.5–12.8% (Table 1). Nonetheless, *T. durum* ‘Purple’ had a higher gluten index (GI of 37 against 31 of the ‘Red’), values that can be considered low in durum wheat [27,28,29]. The GI is a widely accepted parameter to express gluten strength, and it is considered highly inheritable [30]. Hence, the observed difference reveals distinct technological features of the two landraces. Nonetheless, the relationship of the GI with the protein content should not be thought as linear [29,31]. For both varieties, the latter was not far from the upper limit for durum wheat, usually from 7% to 18%, with an average of 12%. The protein content and the gluten strength are the main features of the starting material that determine the quality of pasta [28]. Even so, the protein content is often considered more important than the strength of the gluten, being more closely correlated with the positive features of dried pasta, although this relationship can be affected by the processing method and the gluten composition [32]. The semolina of ‘Purple’ and ‘Red’ had ash contents of 1.25 and 1.35%, respectively. The ash content in the grain is under genetic and environmental control. It is typically influenced by mineral fertilization and positively correlated with the protein content [33]. A low ash content in milled durum grains is considered a quality feature, although it does not significantly affect the industrial performance of the semolina. In Italy, as in other countries, the ash content in common and durum wheat is regulated by law (D.P.R. 9 February 2001, n. 187). The observed values exceed the threshold for “*semola*” and are within limits for “*semolato*”. It should be added that the ash content lowers with sequential debrannings. For instance, in a group of 11 Italian varieties encompassing traditional and contemporary durum wheat cultivars, the ash content reached an acceptable level for “*semola*” after five successive debranning treatments [34].

### 3.2. Polyphenol Content

The grain, semolina, and bran of both ‘Purple’ and ‘Red’ were subjected to an evaluation of the total polyphenol content because these are a wide group of chemically diverse secondary metabolites that are generally known for their positive effects on human health [35,36]. Cereals, as many other plant species, contain different phenolic compounds, and the most important are the derivatives of the cinnamic acid (i.e., coumaric, caffeic, and ferulic acids), flavonoids, and lignans [37]. It is believed that their function is predominantly non-nutritive [37].

The quantification of total polyphenols indicated that the bran of the ‘Purple’ landrace had a significantly higher amount (almost double) of both phenolic fractions, while differences were not significant for the insoluble fractions of the semolina and grains (Table 2).

Specifically, ‘Purple’ bran and grains had an almost double phenolic content (112 and 103 µg/g DM of the equivalents of gallic acid, respectively) compared to those of the ‘Red’ (64 and 65 µg/g DM of gallic acid equivalents, respectively), while the semolina content of ‘Red’ (49 µg/g DM of gallic acid equivalents) was significantly higher than that of the semolina of ‘Purple’ (31 µg/g DM of gallic acid equivalents). The same was true for the insoluble bound phenolic fractions. In ‘Purple’, the phenolic content in the bran and grain was 40% and 23% higher compared to ‘Red’. This can be explained considering that the phenolic content in wheat grains is mainly concentrated in the bran with a limited contribution of the organs and tissue originating the milled semolina. Nonetheless, the two varieties differed in the variation of the ratio insoluble/soluble polyphenols across the analyzed material. This parameter little varied in ‘Purple’ (coefficient of variation, CV: 7.9%), while the CV was 24.3% for ‘Red’, whose semolina had the lowest insoluble/soluble polyphenol ratio.

### 3.3. Determination of Phenolic Acids

HPLC analysis of the semolina revealed several phenolic derivatives, with four compounds (i.e., ferulic, *p*-hydroxybenzoic, vanillic, and *p*-coumaric acids) being the most abundant. The analysis of the two landraces indicated a similar profile in terms of quantity and rank of the chemical compounds, with the ferulic acid always present in a predominant amount, followed, for both varieties, by the vanillic acid or the *p*-coumaric acid in the soluble or insoluble fraction, respectively (Table 3).

In the ‘Red’ variety, the ferulic acid was 639 mg/kg DM and 18.3 mg/kg DM in the insoluble fraction and in the soluble fraction, respectively (Table 3). The ferulic acid is considered the predominant free- and bound-form of polyphenols in cereals, especially (brown) rice and corn [38]. In durum wheat, the ferulic acid contents are slightly influenced by the environment in normal agronomic conditions, and it is mostly influenced by the genotype and altered by abiotic stress [39]. In the soluble fraction, of the three other quantified phenolic acids, the most abundant was vanillic acid, with concentrations in the range of 7.2–7.4 µg/kg DM, followed by *p*-hydroxybenzoic acid, with concentrations in the range of 3.1–3.3 µg/kg DM. The *p*-coumaric acid was the less abundant constituent, with concentrations in the range of 2.2–2.5 µg/kg DM. In the insoluble fraction of the three other quantified phenolic acids, the most abundant was *p*-coumaric acid, with concentrations in the range of 18.5–19.2 µg/kg DM, and with much lower concentrations of vanillic acid (3.1–3.4 µg/kg DM) and *p*-hydroxybenzoic acid (2.1–2.3 mg/kg DM). Overall, the *p*-hydroxybenzoic and the vanillic acids were present in higher quantities in the soluble fractions of the semolina, while the amount of ferulic and *p*-coumaric acids was larger than insoluble fraction. Finally, we did not observe differences between the two varieties in the acids in the soluble and insoluble bound phenolic fraction of the semolina.

### 3.4. Anthocyanins and Proanthocyanidins Content

Anthocyanins and proanthocyanidins (expressed as the micrograms of catechin equivalents per gram of DM) were also quantified in the grain, semolina, and bran of ‘Purple’ and ‘Red’ (Table 4).

The content of anthocyanins in the grain of ‘Purple’ was higher than in ‘Red’, almost double in the bran (72.9 vs. 36.3 µg/g DM) and triple in the grain (116.6 vs. 39.2 µg/g DM) (Table 4). In contrast, the semolina of ‘Red’ contained 25% more anthocyanin than ‘Purple’ (16.8 vs. 12.5 µg/g DM, respectively). A similar trend was observed for proanthocyanidins (polymers or oligomers of anthocyanidin). Their content was 33% higher in the bran of the ‘Purple’ compared to that of the ‘Red’ (1530 vs. 1031 µg/g DM of catechin equivalents, respectively) and 47% more in the grain of the ‘Purple’ than that of the ‘Red’ (3437 vs. 1807 µg/g DM of catechin equivalents, respectively). On the other hand, the amount of proanthocyanidins in the semolina was almost double (+47%) in the ‘Red’ compared to the ‘Purple’ (466 vs. 244 µg/g DM of catechin equivalents, respectively).

Anthocyanins are a class of water-soluble pigments belonging to the flavonoid family. They show a range of pharmacological activities because of their antioxidant and anti-inflammatory properties, with potential therapeutic benefits [40]. In durum wheat, anthocyanins mainly accumulate in the pericarp and aleurone [5]. This may explain why the less colored ‘Red’ variety yielded semolina with a significantly higher content of anthocyanins and proanthocyanidins. Anthocyanin-rich grains can be then used to produce functional foods and considering that these compounds are in the less noble coat of the grain, it has been also proposed that grains can be also exploited as a natural source to extract these pigments [5].

The determination of the anthocyanin compounds by HPLC revealed the presence of significant differences between the material and the variety (Table 5). Overall, the cyanidin 3-glucoside was the most abundant compound, followed by peonidin 3-glucoside. Quantitative differences between the varieties were most pronounced for the bran, with the ‘Purple’ having on average a threefold higher amount than ‘Red’ of the detected molecules. Comparing the material, as expected, anthocyanins were in much lower quantities in the semolina, with the two major anthocyanins detected only in the ‘Purple’ variety. Interestingly, the analysis of the grain revealed both qualitative and quantitative differences. Specifically, the differences in relative terms between the two varieties were more limited compared to the bran. Moreover, only the grain of the ‘Red’ variety contained detectable amounts of delphinidin 3-glucoside and delphinidin 3-rutinoside.

Cyanidin 3-glucoside is often the most abundant anthocyanins in colored cereals (e.g., rice and corn), as well as in most of the plants [40]. For purple common wheat, cyanidin 3-glucoside, peonidin 3-glucoside, and cyanidin 3-galactoside have been described as the most abundant compounds [41,42]. In blue common wheat, cyanidin 3-glucoside is predominant, with pelargonidin 3-glucoside and cyanidin 3-galactoside present in lower amounts [43]. Although intra-varietal differences in colors are sufficiently explained by variation in anthocyanins [44], it is not straightforward to correlate the color of a variety with the type, number, and quantity of pigments, also considering that the influence of the anthocyanins on the plant tissue hue (and tint) is determined by various factors besides their total amount and ratio [45,46]. It is therefore interesting that the grain of the ‘Red’ variety contained delphinidins in a low amount compared to other pigments, while these were not detected in the ‘Purple’. These anthocyanins are typically associated with dark grains [5]. For instance, in a blue common wheat, delphinidin 3-glucoside and delphinidin 3-rutinoside accounted for 69.3% of the detected anthocyanins [42]. Nonetheless, they were also not found in a purple common wheat [42]. In addition to wheats also in purple rye grains cyanidin-3-glucoside is the predominant anthocyanin, followed by peonidin-3-glucoside [47,48] as in our ‘Purple’.

We also quantified major anthocyanidins (i.e., cyanidin, delphinidin, malvidin, peonidin, and petunidin) in the ‘Purple’ and ‘Red’ bran, semolina, and grain (Table 6) because these are typical of colored wheat grains [10]. In grains, cyanidin was the most abundant aglycones for all the material, followed by delphinidins. Other works indicated that cyanidin also the main aglycone in purple common and durum wheat varieties, but it was followed by peonidin [49]. Petunidin was detected in smaller quantities only in the two brans, while peonidin and malvidin only in the grains of the ‘Purple’.

The total anthocyanin content was higher in the ‘Purple’ than in the ‘Red’; precisely, it was just over double of that contained in the bran (25 vs. 12.01 μg/kg DM), more than double of that contained in the grain (12.29 vs. 5.09 μg/kg DM), and almost 10 times that contained in the semolina (5.36 vs. 0.56 μg/kg DM).

### 3.5. Antioxidant Activity

Tests for the evaluation of antioxidant activity show the highest values for the grain in both varieties compared to bran and semolina. Moreover, in both assays the highest values were observed for the ‘Purple’ variety compared to that ‘Red’ (Table 7). Mean values were not significantly different between the semolina of the two varieties. In the brans, were higher values in the DPPH test (respectively, ABTS test) were recorded for the ‘Purple’ (resp. ‘Red’). As previously reported [50], the highest antioxidant capacity was observed in whole grains, which contain more bioactive compounds of health interest, such as insoluble fiber, phenolic acids, and alkylresorcinols [51].

## 4. Conclusions

The characterization of *Triticum durum* ‘Purple’ and ‘Red’, two varieties imported from Ethiopia and grown in southern Italy, highlighted their distinctive features such as an above-average protein content, which should positively influence the pasta-making quality. The ferulic acid was particularly abundant among phenolic acids in both the soluble and insoluble phenolic fractions of the grain, semolina, and bran. Even if related to the analysis of two landraces, our work also revealed the qualitative and quantitative diversity in anthocyanin content in durum wheat. It should be added that the material under investigation derives from a non-mass-production system in which grains are processed with a multi-pass methodology using a rolling mill. These systems generate less heat during grinding also because multiple passes allow the achievement of size reduction more gradually [52]. In the future, it may be worth investigating to what extent the high antioxidant activities in the semolina may be affected by the milling method. The lower gluten index, high protein level, rich and composite range of secondary metabolites, along with the antioxidant activities, indicate that the germplasm under investigation has interesting features for the niche market of functional durum wheat products in specific geographical areas as an alternative to the mass-produced Italian goods required for international markets.

## Figures and Tables

**Table 1:** Protein, dry gluten and ash content and gluten index in semolina of durum wheat ‘Purple’ and ‘Red’ landraces. Values are reported as mean ± SD (*n* = 5). Significant differences between the two varieties for bran, semolina, or grain are indicated by lowercase letters (*t*-test, *p* < 0.05).

Parameters (%)	‘Purple’	‘Red’
Protein	15.20 ± 0.10 b	16.80 ± 0.20 a
Dry gluten	12.80 ± 0.88	12.50 ± 0.06
Ash	1.25 ± 0.02 b	1.35 ± 0.01 a
Gluten index	37.00 ± 0.20 a	31.00 ± 0.60 b

**Table 2:** Soluble and insoluble polyphenolic fraction in ‘Purple’ and ‘Red’ landraces. Values are reported as mean ± SD (*n* = 5). Significant differences between the two varieties for bran, semolina, or grain are indicated by lowercase letters (*t*-test, *p* < 0.05).

Polyphenols (µg/g DM Gallic Acid Equivalent)	Bran		Semolina		Grain	
	‘Purple’	‘Red’	‘Purple’	‘Red’	‘Purple’	‘Red’
Soluble phenolic fraction	112 ± 21 a	64 ± 4 b	31 ± 4 b	49 ± 3 a	103 ± 15 a	65 ± 5 b
Insoluble bound phenolic fraction	2391 ± 471 a	1430 ± 284 b	751 ± 105	849 ± 112	2380 ± 455	1840 ± 329

**Table 3:** Identification and quantification of the acids in the soluble and insoluble bound phenolic fraction in semolina of ‘Purple’ and ‘Red’ based on retention times and calibration lines for comparison with the corresponding commercial products. Values are reported as mean ± SD (*n* = 5). Mean values between the two varieties were not statistically different (*t*-test, *p* ≥ 0.05).

Soluble Hpenolic Fraction (mg/kg DM)	‘Purple’	‘Red’
Ferulic acid	17.9 ± 1.10	18.3 ± 1.05
*p*-Hydroxybenzoic acid	3.1 ± 0.28	3.3 ± 0.27
Vanillic acid	7.2 ± 0.33	7.4 ± 0.38
*p*-Coumaric acid	2.2 ± 0.32	2.5 ± 0.30
**Insoluble Bound Phenolic Fraction** **(mg/kg DM)**		
Ferulic acid	625 ± 39.3	639 ± 41.4
*p*-Hydroxybenzoic acid	2.1 ± 0.29	2.3 ± 0.28
Vanillic acid	3.1 ± 0.35	3.4 ± 0.29
*p*-Coumaric acid	18.5 ± 2.35	19.2 ± 2.26

**Table 4:** Anthocyanins and proanthocyanidins in the ‘Purple’ and ‘Red’ landraces. Values are reported as mean ± SD (*n* = 5). Significant differences between the two varieties for bran, semolina, or grain are indicated by lowercase letters (*t*-test, *p* < 0.05).

Phenolic Components (µg/g DM Catechin Equivalent)	Bran		Semolina		Grain	
	‘Purple’	‘Red’	‘Purple’	‘Red’	‘Purple’	‘Red’
Anthocyanins	72.9 ± 1.1 a	36.3 ± 0.4 b	12.5 ± 0.8 b	16.8 ± 0.1 a	116.6 ± 1.9 a	39.2 ± 0.8 b
Proanthocyanidins	1530 ± 133 a	1031 ± 33 b	244 ± 22 b	466 ± 111 a	3437 ± 377 a	1807 ± 166 b

**Table 5:** Anthocyanin composition in the ‘Purple’ and ‘Red’ landraces (mean ± SD; *n* = 5) determined by HPLC. Values are reported as mean ± SD (*n* = 5). Significant differences between the two varieties for bran, semolina, or grain are indicated by lowercase letters (*t*-test, *p* < 0.05).

Anthocyanins (µg/kg DM)	Bran		Semolina		Grain	
	‘Purple’	‘Red’	‘Purple’	‘Red’	‘Purple’	‘Red’
Delphinidin 3-glucoside	-	-	-	-	-	1.20 ± 0.10
Delphinidin 3-rutinoside	-	-	-	-	-	2.66 ± 0.14
Cyanidin 3-glucoside	5.09 ± 0.11 a	1.15 ± 0.05 b	2.05 ± 0.15	-	7.32 ± 0.48 a	2.39 ± 0.03 b
Petunidin 3-glucoside	1.53 ± 0.11 a	0.57 ± 0.07 b	-	-	1.55 ± 0.05 a	1.29 ± 0.01 b
Peonidin 3-glucoside	2.05 ± 0.05 a	0.86 ± 0.06 b	0.89 ± 0.05	-	3.35 ± 0.15 a	1.68 ± 0.04 b
Malvidin 3-glucoside	-	-	-	-	-	-

**Table 6:** Anthocyanidin composition in the ‘Purple’ and ‘Red’ (mean ± SD; *n* = 5) determined by HPLC. Values are reported as mean ± SD (*n* = 5). Significant differences between the two varieties for bran, semolina, or grain are indicated by lowercase letters (*t*-test, *p* < 0.05).

Anthocyanidins (µg/kg DM)	Bran		Semolina		Grain	
	‘Purple’	‘Red’	‘Purple’	‘Red’	‘Purple’	‘Red’
Delphinidin	1.75 ± 0.05 a	0.91 ± 0.01 b	1.15 ± 0.05	-	3.15 ± 0.45 a	0.87 ± 0.03 b
Cyanidin	1.87 ± 0.03	1.60 ± 0.20	1.27 ± 0.07 a	0.56 ± 0.14 b	5.45 ± 0.35 a	1.47 ± 0.03 b
Petunidin	*-*	*-*	*-*	*-*	1.68 ± 0.08 a	0.45 ± 0.05 b
Peonidin	*-*	*-*	*-*	*-*	1.35 ± 0.05	*-*
Malvidin	*-*	*-*	*-*	*-*	1.15 ± 0.75	*-*
Total anthocyanins (Anthocyanins + Proanthocyanidins)	12.29 ± 0.15 a	5.09 ± 0.37 b	5.36 ± 0.32 a	0.56 ± 0.14 b	25.00 ± 0.40 a	12.01 ± 0.09 b

**Table 7:** Antioxidant activity (μmol Trolox equivalent/g DM) in ‘Purple’ and ‘Red’ landraces. Values are reported as mean ± SD (*n* = 5). Significant differences between the two varieties for bran, semolina, or grain are indicated by lowercase letters (*t*-test, *p* < 0.05).

Assay	Bran		Semolina		Grain	
	‘Purple’	‘Red’	‘Purple’	‘Red’	‘Purple’	‘Red’
DPPH	9.95 ± 0.30 a	8.47 ± 0.44 b	8.14 ± 0.04	8.03 ± 0.01	11.07 ± 0.59 a	9.85 ± 0.01 b
ABTS	1.75 ± 0.30 b	2.70 ± 0.12 a	1.67 ± 0.24	1.68 ± 0.10	5.29 ± 0.25 a	4.33 ± 0.40 b

## Data Availability

The data are contained within the article.

## References

[1] Hasanuzzaman M., Nahar K., Hossain M.A. (2019). Wheat Production in Changing Environments.

[2] Senatore M., Ward T., Cappelletti E., Beccari G., McCormick S., Busman M., Laraba I., O‘Donnell K., Prodi A. (2021). Species diversity and mycotoxin production by members of the *Fusarium tricinctum* species complex associated with *Fusarium* head blight of wheat and barley in Italy. Int. J. Food Microbiol..

[3] Lupton F. (2014). Wheat Vreeding: Its Scientific Basis.

[4] Royo C., Nachit M.M., Di Fonzo N., Araus J.L., Pfeiffer W.H., Slafer G.A. (2005). Durum Wheat Breeding: Current Approaches and Future Strategies, Volumes 1 and 2.

[5] Ficco D.B., Mastrangelo A.M., Trono D., Borrelli G.M., De Vita P., Fares C., Beleggia R., Platani C., Papa R. (2014). The colours of durum wheat: A review. Crop Pasture Sci..

[6] Arzani A., Ashraf M. (2017). Cultivated ancient wheats (*Triticum* spp.): A potential source of health-beneficial food products. Comp. Rev. Food Sci. Food Safety.

[7] Cooper R. (2015). Re-discovering ancient wheat varieties as functional foods. J. Tradit. Complementary Med..

[8] Dinu M., Whittaker A., Pagliai G., Benedettelli S., Sofi F. (2018). Ancient wheat species and human health: Biochemical and clinical implications. J. Nutr. Biochem..

[9] Shoeva O.Y., Gordeeva E.I., Khlestkina E.K. (2014). The regulation of anthocyanin synthesis in the wheat pericarp. Molecules.

[10] Lachman J., Martinek P., Kotíková Z., Orsák M., Šulc M. (2017). Genetics and chemistry of pigments in wheat grain–A review. J. Cereal Sci..

[11] Brandolini A., Hidalgo A., Gabriele S., Heun M. (2015). Chemical composition of wild and feral diploid wheats and their bearing on domesticated wheats. J. Cereal Sci..

[12] Yilmaz V.A., Brandolini A., Hidalgo A. (2015). Phenolic acids and antioxidant activity of wild, feral and domesticated diploid wheats. J. Cereal Sci..

[13] Newton A.C., Akar T., Baresel J.P., Bebeli P.J., Bettencourt E., Bladenopoulos K.V., Czembor J.H., Fasoula D.A., Katsiotis A., Koutis K. (2011). Cereal landraces for sustainable agriculture. Sustain. Agric..

[14] AACC (2005). Method 38-12.02.

[15] Hayes M. (2020). Measuring protein content in food: An overview of methods. Foods.

[16] Ismail B.P. (2017). Ash content determination. Food Analysis Laboratory Manual.

[17] Abdel-Aal E.-S., Hucl P., Sosulski F., Graf R., Gillott C., Pietrzak L. (2001). Screening spring wheat for midge resistance in relation to ferulic acid content. J. Agric. Food Chem..

[18] Singleton V.L., Orthofer R., Lamuela-Raventós R.M. (1999). [14] Analysis of total phenols and other oxidation substrates and antioxidants by means of folin-ciocalteu reagent. Methods Enzymol..

[19] Bastola K.P., Guragain Y.N., Bhadriraju V., Vadlani P.V. (2017). Evaluation of standards and interfering compounds in the determination of phenolics by Folin-Ciocalteu assay method for effective bioprocessing of biomass. Am. J. Anal. Chem..

[20] Medina M.B. (2011). Simple and rapid method for the analysis of phenolic compounds in beverages and grains. J. Agric. Food Chem..

[21] Dellagreca M., Fiorentino A., Izzo A., Napoli F., Purcaro R., Zarrelli A. (2007). Phytotoxicity of secondary metabolites from *Aptenia cordifolia*. Chem. Biodivers..

[22] Hosseinian F.S., Li W., Beta T. (2008). Measurement of anthocyanins and other phytochemicals in purple wheat. Food Chem..

[23] Siebenhandl S., Grausgruber H., Pellegrini N., Del Rio D., Fogliano V., Pernice R., Berghofer E. (2007). Phytochemical profile of main antioxidants in different fractions of purple and blue wheat, and black barley. J. Agric. Food Chem..

[24] Sun B., Ricardo-da-Silva J.M., Spranger I. (1998). Critical factors of vanillin assay for catechins and proanthocyanidins. J. Agric. Food Chem..

[25] Lavelli V., Hidalgo A., Pompei C., Brandolini A. (2009). Radical scavenging activity of einkorn (*Triticum monococcum* L. subsp. monococcum) wholemeal flour and its relationship to soluble phenolic and lipophilic antioxidant content. J. Cereal Sc..

[26] Re R., Pellegrini N., Proteggente A., Pannala A., Yang M., Rice-Evans C. (1999). Antioxidant activity applying an improved ABTS radical cation decolorization assay. Free Radic. Biol. Med..

[27] Vida G., Szunics L., Veisz O., Bedő Z., Láng L., Árendás T., Bónis P., Rakszegi M. (2014). Effect of genotypic, meteorological and agronomic factors on the gluten index of winter durum wheat. Euphytica.

[28] Ames N., Clarke J., Marchylo B., Dexter J., Woods S. (1999). Effect of environment and genotype on durum wheat gluten strength and pasta viscoelasticity. Cereal Chem..

[29] Oikonomou N., Bakalis S., Rahman M., Krokida M. (2015). Gluten index for wheat products: Main variables in affecting the value and nonlinear regression model. Int. J. Food Prop..

[30] Clarke F., Clarke J., Pozniak C., Knox R., McCaig T. (2009). Protein concentration inheritance and selection in durum wheat. Can. J. Plant Sci..

[31] Cecchini C., Bresciani A., Menesatti P., Pagani M.A., Marti A. (2021). Assessing the rheological properties of durum wheat semolina: A Review. Foods.

[32] Ames N., Clarke J., Marchylo B., Dexter J., Schlichting L., Woods S. (2003). The effect of extra-strong gluten on quality parameters in durum wheat. Can. J. Plant Sci..

[33] Peterson C. (1986). Influence of cultivar and environment on mineral and protein concentrations of wheat flour, bran, and grain. Cereal Chem..

[34] Fares C., Troccoli A., Di Fonzo N. (1996). Use of friction debranning to evaluate ash distribution in Italian durum wheat cultivars. Cereal Chem..

[35] Dykes L., Rooney L. (2007). Phenolic compounds in cereal grains and their health benefits. Cereal Foods World.

[36] Hodzic Z., Pasalic H., Memisevic A., Srabovic M., Saletovic M., Poljakovic M. (2009). The influence of total phenols content on antioxidant capacity in the whole grain extracts. Eur. J. Sci. Res..

[37] Salunkhe D., Jadhav S., Kadam S., Chavan J., Luh B. (1983). Chemical, biochemical, and biological significance of polyphenols in cereals and legumes. Crit. Rev. Food Sci. Nutr..

[38] Van Hung P. (2016). Phenolic compounds of cereals and their antioxidant capacity. Crit. Rev. Food Sci. Nutr..

[39] Lafiandra D., Masci S., Sissons M., Dornez E., Delcour J., Courtin C., Caboni M.F., Sissons M., Marchylo B., Abecassis J. (2012). Kernel components of technological value. Durum Wheat Chemistry and Technology.

[40] Khoo H.E., Azlan A., Tang S.T., Lim S.M. (2017). Anthocyanidins and anthocyanins: Colored pigments as food, pharmaceutical ingredients, and the potential health benefits. Food Nutr. Res..

[41] Abdel-Aal E.-S.M., Hucl P. (2003). Composition and stability of anthocyanins in blue-grained wheat. J. Agric. Food Chem..

[42] Abdel-Aal E.-S.M., Young J.C., Rabalski I. (2006). Anthocyanin composition in black, blue, pink, purple, and red cereal grains. J. Agric. Food Chem..

[43] Hu C., Cai Y.-Z., Li W., Corke H., Kitts D.D. (2007). Anthocyanin characterization and bioactivity assessment of a dark blue grained wheat (*Triticum aestivum* L. cv. Hedong Wumai) extract. Food Chem..

[44] Biolley J., Jay M. (1993). Anthocyanins in modern roses: Chemical and colorimetric features in relation to the colour range. J. Exp. Bot..

[45] Fossen T., Cabrita L., Andersen O.M. (1998). Colour and stability of pure anthocyanins influenced by pH including the alkaline region. Food Chem..

[46] Torskangerpoll K., Andersen Ø.M. (2005). Colour stability of anthocyanins in aqueous solutions at various pH values. Food Chem..

[47] Dedio W., Hill R., Evans L. (1972). Anthocyanins in the pericarp and coleoptiles of purple wheat. Can. J. Plant Sc..

[48] Dedio W., Hill R., Evans L. (1972). Anthocyanins in the pericarp and coleoptiles of purple-seeded rye. Can. J. Plant Sc..

[49] Abdel-Aal E.S.M., Hucl P., Shipp J., Rabalski I. (2016). Compositional differences in anthocyanins from blue-and purple-grained spring wheat grown in four environments in Central Saskatchewan. Cereal Chem..

[50] Cammerata A., Laddomada B., Milano F., Camerlengo F., Bonarrigo M., Masci S., Sestili F. (2021). Qualitative characterization of unrefined durum wheat air-classified fractions. Foods.

[51] Hemery Y., Rouau X., Lullien-Pellerin V., Barron C., Abecassis J. (2007). Dry processes to develop wheat fractions and products with enhanced nutritional quality. J. Cereal Sci..

[52] Prabhasankar P., Haridas Rao P. (2001). Effect of different milling methods on chemical composition of whole wheat flour. Eur. Food Res. Technol..

